# Development and usability of a mobile ecological momentary assessment platform for dietary surveillance in the U.S.

**DOI:** 10.1186/s12966-026-01916-x

**Published:** 2026-04-25

**Authors:** Susan M. Schembre, Michelle R. Jospe, Rick Weiss, Christopher A. Taylor, Edward J. Bedrick, Jessie Somerville, Julia Felrice, Kelli M. Richardson, Genevieve F. Dunton, Cynthia A. Thomson

**Affiliations:** 1 Department of Oncology, Lombardi Comprehensive Cancer Center, Georgetown University, 2115 Wisconsin Avenue NW Suite 300, Washington, DC 20007 USA; 2 Viocare, Inc., Princeton, NJ 08542 USA; 3 Division of Medical Dietetics, The Ohio State University, Columbus, OH 43210 USA; 4 Department of Epidemiology and Biostatistics, Mel and Enid Zuckerman College of Public Health, University of Arizona, Tucson, AZ 85724 USA; 5 Department of Population and Public Health Sciences, University of Southern California, Los Angeles, CA 90039 USA; 6 Department of Health Promotion Sciences, Mel and Enid Zuckerman College of Public Health, University of Arizona, Tucson, AZ 85724 USA

**Keywords:** Ecological momentary assessment, Dietary surveillance, Mobile health, User-centered design, Usability testing, Dietary measurement

## Abstract

**Background:**

Dietary surveillance is critical for addressing diet-related chronic diseases, yet traditional methods like 24-hour dietary recalls and food frequency questionnaires suffer from recall bias, high respondent burden, and inaccurate portion size estimation. Mobile ecological momentary dietary assessment (*mEMDA*) enables real-time dietary capture, reducing memory load and minimizing reactivity. This study describes the user-centered development and iterative usability testing of *Edna*, a research-quality mEMDA platform for large-scale, low-burden dietary surveillance. *Edna* integrates real-time self-report with an image-supported, demographically inclusive food list derived from NHANES data and automated linkage to USDA nutrient databases. Here, we describe *Edna’s* development and usability in diverse U.S. adults.

**Methods:**

Three sequential rounds of iterative, user-centered development and usability testing were conducted between June 2024 and June 2025. Participants (*N* = 146 U.S. adults, ages 19–65 years) were recruited nationally through recruited nationally through an online research volunteer registry using stratified sampling by age, sex, BMI, and geographic region. Participants were randomly assigned to event-contingent or interval-contingent sampling conditions and asked to log all dietary intake for 14 consecutive days. Development refinements were guided by quantitative usability metrics and structured participant feedback through daily surveys and end-of-study questionnaires between rounds. Primary outcomes included System Usability Scale (*SUS*) scores, percentage of days with logged entries (engagement), and interval completion (interval-contingent only). The app was iteratively refined between rounds based on user feedback until SUS scores plateaued (change < 3 points). Statistical analyses included independent samples t-tests and ANOVA.

**Results:**

SUS scores increased from 71.7 (SD = 23.0) in Round 1 to 78.8 (SD = 19.3) in Round 3, exceeding the digital health app benchmark of 68. Engagement ranged from 92.5 to 97.6% of study days, and interval completion among interval-contingent users ranged from 76 to 87%. Participants rated portion-size images as clear and culturally inclusive, with 94% reporting excellent overall experience.

**Conclusions:**

*Edna* achieved above-average usability and engagement, demonstrating that mEMDA is acceptable for population-level dietary surveillance. These findings support integration of EMA-based tools into routine public health nutrition monitoring to capture dietary behaviors more dynamically with reduced burden compared to traditional assessment methods.

**Supplementary Information:**

The online version contains supplementary material available at 10.1186/s12966-026-01916-x.

## Background

Dietary surveillance is critical to U.S. public-health efforts because diet-related chronic diseases, including obesity, type 2 diabetes, cardiovascular disease, and several cancers, remain leading causes of preventable morbidity and mortality [1, 2]. National dietary data serve as exposure measures for etiologic research, calibrate measurement error, enable dose-response and risk assessments, validate dietary biomarkers, and support impact evaluations and policy counterfactuals [3, 4]. Although emerging objective and non–self-report dietary assessment approaches, such as image-based methods, wearable sensors, and metabolomics, offer promise, they remain inaccessible and insufficiently validated for use in population-level surveillance [5–8]. Accordingly, public health surveillance will continue to rely on modernized self-report tools capable of producing high-quality, scalable dietary data.

Traditional dietary surveillance tools such as 24-hour dietary recalls (*24 h*) and food frequency questionnaires (*FFQ*) are widely used due to their practicality and scalability but are limited by recall bias, social desirability bias, respondent burden, and inaccuracies in portion-size estimation, particularly in diverse populations [9–11]. Ecological momentary assessment (*EMA*) offers a strategy whereby individuals complete brief, repeated surveys in real or near real-time using mobile devices [12]. When applied to diet, mobile ecological momentary dietary assessment (*mEMDA*) shortens recall intervals, reduces memory load, and enables contextual data capture while supporting automation and scalability [13]. Despite these advantages, mEMDA tools and sampling strategies have not yet been systematically optimized or validated for population-level dietary surveillance, and few have been evaluated specifically for usability and engagement as surveillance tools [13, 14].

Recent advances in mobile health technology have created new opportunities for near real-time, low-cost dietary data collection at scale [15]. Supporting evidence for mEMDA feasibility and acceptability is accumulating across age groups and use cases, with reported prompt response rate typically ranging from 75% to 80% [13, 16, 17]. Additional recent studies have demonstrated feasibility in diverse contexts, including experience sampling-based intake assessment, monitoring of online food delivery behaviors, microtemporal tracking of diet and related factors, and eating rhythm self-monitoring [18–21]. Collectively, this literature supports the promise of EMA-based dietary assessment but has primarily focused on short-term monitoring or specific behavioral applications rather than scalable, population-level surveillance.

Building on this foundation, we developed *Edna*, a research quality, EMA-based Diet and Nutrition Assessment platform. Design priorities included minimizing user burden, shortening recall intervals, improving portion-size estimation, and ensuring accessibility across diverse populations. *Edna* integrates real-time self-report with a demographically inclusive food list derived from nationally representative intake data and automated linkage to a publicly available nutrient database. This paper describes *Edna’s* data-driven, user-centered development and reports findings from an iterative usability study conducted to refine the platform’s interface and feature set prior to validation testing.

## Methods

### Conceptual rationale for *Edna*

Building on prior research and the need for scalable surveillance tools, *Edna* was designed to operationalize mEMDA within a framework compatible with National Health and Nutrition Examination Survey (*NHANES*) parameters for U.S. adults aged 19 to 65 years. Rather than replicate traditional recall structures, the platform translates surveillance requirements into a hierarchical, mobile-first architecture that integrates structured food selection, streamlined meal-entry pathways, image-assisted portion estimation, and automated nutrient coding through publicly available composition databases. Population-level intake alignment was informed by analyses of nationally representative dietary data to ensure consistency with consumption patterns across demographic subgroups, with deliberate attention to inclusivity and usability. The current proof-of-concept version of *Edna* focuses on saturated fat and added sugar as sentinel nutrients of public health relevance while establishing the infrastructure for broader nutrient expansion.

### Technical specifications

*Edna* was developed in partnership with Viocare, Inc., a National Institutes of Health (*NIH*)-funded diet-assessment software company. The mobile application was built using the React Native Expo framework to support cross-platform deployment for iOS and Android. Platform-specific builds are distributed through the Apple App Store and Google Play. The backend infrastructure is hosted on Firebase. User authentication is managed through Google Sign-In. Participant data are stored in Firestore, a NoSQL database, with Cloud Functions supporting background processes such as notification scheduling and data processing. Firebase Storage manages user-generated content with access controls enforced through Firebase security rules. Dietary entries are stored using unique food codes mapped to NHANES-based food items and recipes through the USDA Food and Nutrient Database for Dietary Studies (*FNDDS*), enabling generation of time-stamped nutrient output files. A secure, web-based administrative dashboard provides role-based access for participant management, engagement monitoring, system logs, and data export. The dashboard is built with React JS and integrated with the same backend infrastructure as the mobile app.

### Food list

*Edna’s* food list was developed using a structured, data-driven approach to support precise dietary assessment among U.S. adults. Saturated fat and added sugar were selected as initial target nutrients. Using NHANES 2005–2018 data, we identified leading contributors to these nutrients by aggregating two days of 24-hour dietary recall data from more than 36,000 adults [22]. Reported foods were mapped to the 150 + USDA “What We Eat in America” third-level food categories (e.g., 1st level: Grains, 2nd level: Ready-to-eat cereals, 3rd level: Ready-to-eat higher sugar cereals) and ranked by proportional contribution to saturated fat and added sugar intake in the overall population and across NHANES-defined sociodemographic subgroups. Categories contributing to the top 90% of intake were retained, resulting in 95 third-level food categories that captured ≥ 90% of targeted nutrient intake for more than 88% of U.S. adults [22].

To prioritize foods most relevant to daily intake estimation, we applied a 2-gram inclusion threshold by retaining foods that typically contribute at least 2 g of saturated fat or added sugars per 100 g. This cutoff was derived from dietary guidelines recommending no more than 10% of daily energy from saturated fat (~ 20 g/day on a 2,000-kcal diet) [23], allowing us to focus on items with a meaningful nutritional impact. In cases where a nutrient-dense food (e.g., cheese) was commonly consumed with a low-nutrient “vehicle” food (e.g., crackers), we included both to support accurate meal reconstruction, even when the vehicle food alone fell below the threshold.

The resulting food list was organized into a hierarchical structure comprising 12 broad food groups (Level 1), approximately 350 subgroups (Level 2), and more than 1,000 specific food types (Level 3) [22]. All entries were ultimately linked to FNDDS nutrient profiles through a structured database, enabling real-time nutrient coding and minimizing post-processing burden.

### User interface and user experience design (UI/UX)

#### Sampling protocols by condition

*Edna* supports both event-contingent and interval-contingent dietary reporting workflows. Event-contingent designs prompt self-initiated reporting at the occurrence of a defined event, whereas interval-contingent designs rely on structured, time-based prompts delivered at predefined intervals. An overview of key similarities and differences between the two workflows is provided in Table 1.

**Table 1:** Overview of event- and interval-contingent sampling protocols

Feature	Interval-Contingent	Event-Contingent
Logging trigger	Scheduled prompts every 2.5 h during waking hours, with exception of the final prompt (30 min before sleep)	Participant-initiated at each eating occasion
Prompt expiration	Prompts expire after one day; entries may be logged to minimize data loss	Not applicable
Reminder structure	Not applicable	Optional user-configured reminders; participants may create customizable in-app notifications
Nighttime intake capture	Reported at first prompt of following day	Logged when participant initiates entry
Editing capability	Entries from only the current or previous day can be added and edited	Same

In the interval-contingent workflow, scheduled prompts are delivered every 2.5 h during waking hours asking whether any food or beverage was consumed since the prior interval. The 2.5-hour interval was informed by NHANES meal timing data [24]. The home screen displays prior, current, and upcoming intervals with their corresponding time ranges. If users indicate intake occurred, they are guided through the food entry workflow; if not, the app records no intake. Unlike traditional EMA, prompts do not expire after a brief window, allowing later entry to reduce potential data loss. In the event-contingent workflow, users initiate entries at each eating occasion using meal-type tiles displayed on the home screen. No scheduled prompts are delivered. Instead, users may configure optional in-app reminders at preferred times (e.g., around usual mealtimes). Both workflows include an in-app calendar that allows users to review and edit entries from the current or previous day. Entries prior to the previous day cannot be added or edited. All entries are time-stamped at both consumption and entry, enabling calculation of recall intervals and preserving temporal information for analytic evaluation.

#### Food entry and portion size selection

**Fig. 1:**
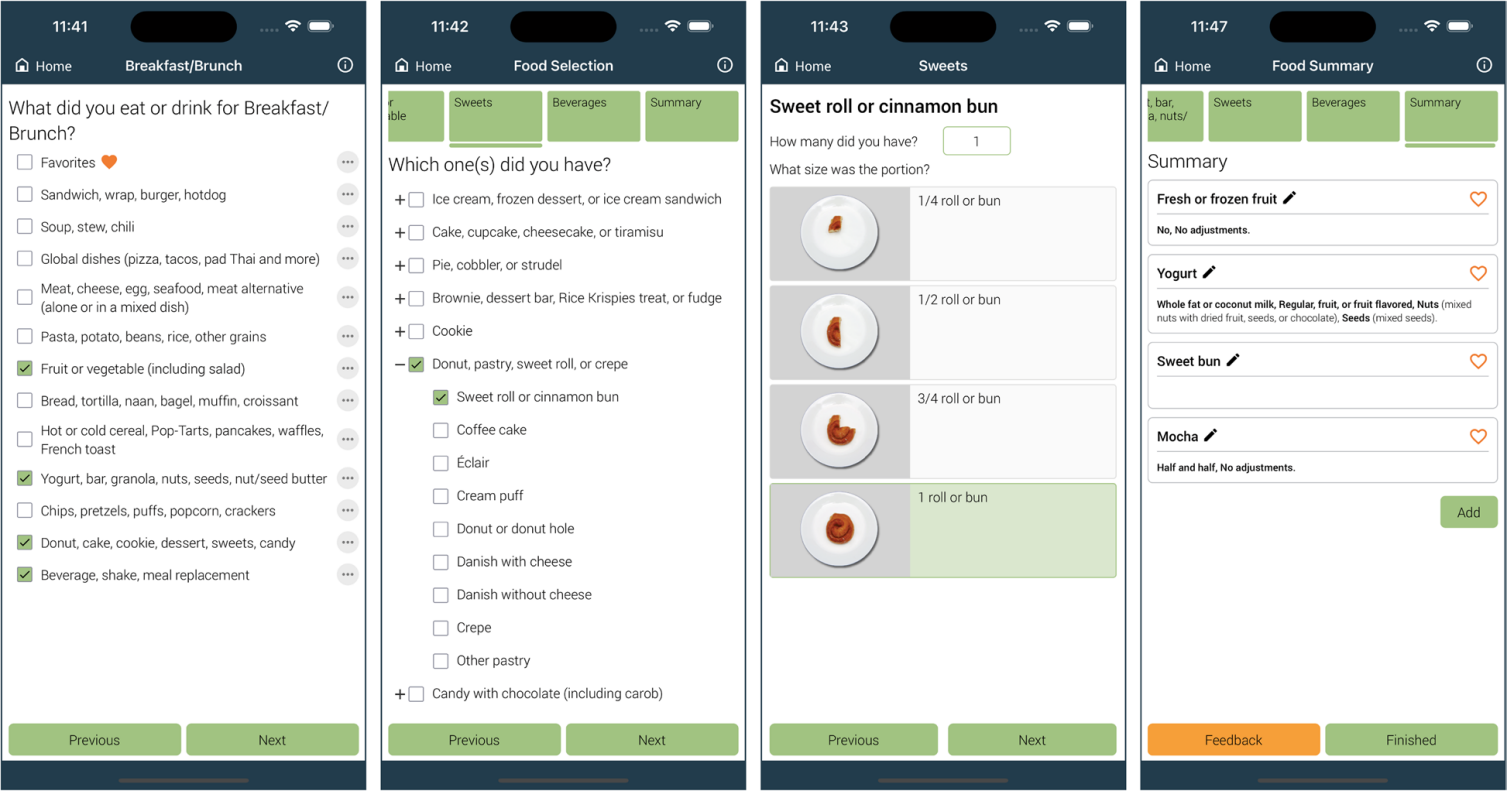
Screenshots of *Edna’s* food logging interface

Edna’s food logging interface uses a structured, hierarchical workflow (Fig. 1). After selecting a meal label (event-contingent) or time interval (interval-contingent) and entering a meal time, users enter foods and beverages consumed, organized as described above, from broad food groupings to increasingly specific options within a nested selection pathway to reduce cognitive load and streamline navigation. Users then specify relevant additions or condiments.

All primary foods and beverages and additions require portion size selection. Portion size is estimated using labeled photographs tailored to each item, adapted from Viocare’s Vioscreen FFQ, a validated web-based dietary assessment tool [25]. For foods with a standard portion (e.g. eggs), a single labeled image is shown and users report the number of servings consumed. For foods without a standard serving size (e.g. casseroles), multiple labeled portion images are displayed in a scrollable format, and users select the image that best reflects the amount consumed. Completed entries appear on a summary screen displaying the selected food, preparation details, and portion size. Users may edit entries or add additional items before final submission.

Features Supporting Reporting *Favorite foods*. Users can mark frequently consumed or leftover items as “Favorites”, which are then stored as a Level 1 food category for quick access in future logging sessions (Fig. 1). Favorite foods retain all previously specified details, including portion size, but can be edited as needed before submission.

*Navigation assistance*. *Edna* offers a built-in “Find My Food” feature. This tool provides step-by-step navigation instructions to help users locate items by name, food group, or common alias. Alias terms were generated using ChatGPT (GPT-4, OpenAI) to account for regional, cultural, and colloquial naming variations. For example, searching for “pop” directs users to soft drinks, and “Pad Thai” links to the broader category of Asian noodle dishes.

#### Onboarding and in-app support

Upon first login, users are guided through onboarding screens introducing the app layout, navigation flow, logging procedures, and prompt response expectations. The tutorial remains accessible via the Help menu. Additional in-app support includes video demonstrations of the logging process, a searchable FAQ section, and a Contact Us feature that enables users to reach the study team or developers for assistance.

### Usability testing and iterative UI/UX refinement

#### Study design and setting

We used a sequential, multimethod design to iteratively evaluate and refine *Edna*. Usability testing was conducted remotely in the U.S. between June 2024 and June 2025, with each round involving 14 days of app use per participant. The study design was intentionally iterative, allowing the research team to modify the app between rounds based on user feedback and performance metrics. Usability testing was conducted until usability scores plateaued, defined as a change of < 3 points between rounds, with a maximum of three rounds. The study was approved by the Georgetown University Institutional Review Board (IRB ID: STUDY00007754).

#### Study participants and randomization

Adults aged 19 to 65 years residing in the U.S., were recruited remotely through ResearchMatch, a volunteer registry supported by the U.S. NIH as part of the Clinical Translational Science Award program (https://www.researchmatch.org/researchers/faq). Volunteers who expressed interest were contacted by study staff via email. Recruitment was stratified to ensure demographic diversity across key characteristics, including sex, age group (19–35, 36–50, 51–65), BMI category (18.5–24.9, 25–29.9, ≥ 30), and U.S. geographic region (Northeast, Southeast, Midwest, Southwest, West). Stratification was used to ensure broad geographic and demographic representation, with a minimum of five participants targeted from each region per round.

Eligible individuals were required to own a smartphone capable of downloading and running the *Edna* app and be able to read and understand English. Individuals were excluded if they reported a current or past diagnosis of an eating disorder or a BMI < 18.5 kg/m², based on self-reported height and weight. Although the study did not target vulnerable populations, individuals from a range of socioeconomic and demographic backgrounds were eligible to participate. Prospective participants were screened for eligibility using REDCap (Research Electronic Data Capture), a secure, web-based software platform designed to support data capture for research studies. Informed consent was obtained electronically prior to participation. All participant characteristic data were collected data were collected during eligibility screening or via a baseline survey following electronic informed consent via REDCap. Following electronic informed consent, participants were enrolled remotely and randomly assigned to one of two sampling conditions: event-contingent or interval-contingent.

#### Procedures

##### *Edna* food entry workflow usability

Participants were instructed to download the *Edna* app from the Apple App Store or Google Play Store and were provided with a study-specific username and a unique password to register the app. Once registered, participants could begin logging food and beverage intake using the flow corresponding to their assigned sampling condition. Study staff monitored the administrative dashboard to confirm successful registration and followed up with participants who had not registered within the first 48 h. Participants who failed to register were contacted up to three times with reminders and troubleshooting queries. Only those who registered the app were able to participate in study activities.

Participants were asked to log all meals, snacks, and beverages consumed over a 14-day period. A 14-day use period was selected to assess short-term engagement under naturalistic conditions and to explore whether participants would continue using *Edna* beyond the typical 3- to 10-day duration previously used in mEMDA studies [13]. No additional training or support was provided beyond the onboarding and help features embedded within the app, allowing for a realistic assessment of standalone usability.

Technical issues were reported by participants through the in-app feedback form, daily REDCap surveys, or direct, email communication with the study team including Viocare personnel. Study staff monitored participant activity daily during the 14-day period using the *Edna* administrative dashboard. App bugs, crashes, and troubleshooting requests were addressed in real time, with any necessary updates deployed without requiring reinstallation. This allowed participants to continue logging without disruption while enabling ongoing refinement of the app between testing rounds.

Structured usability feedback was also collected at three time points using custom REDCap surveys. On the day of registration, participants completed an onboarding feedback form that prompted open-ended reflections on their experience downloading and setting up the app under multiple feedback categories (“Downloading the app,” “Tutorials/Videos,” “Bug reports”) and submitted additional comments as option, open text. During the 14-day use period, participants received daily feedback forms via text, which asked them to categorize their feedback (e.g., “Missing food,” “Suggestions for improvement,” “Bug report”) and provide open-ended comments about their experience that day. These reports offered real-time insight into usability challenges and supported early identification of friction points in the logging workflow.

On day 15, participants were asked to complete end-of-study feedback surveys, including the System Usability Scale (*SUS*). This included a comprehension check about *Edna’s* intended purpose, followed by Likert-style items on the usefulness of the onboarding materials and support features. Open-ended questions captured what participants liked and disliked, identified foods that were difficult to enter, and gathered suggestions for improving the search and entry experience. Users were reminded to complete each of the end of study tasks at least three times before they were considered lost-to-follow-up. The same 14-day protocol was used for all rounds. After each round, participant feedback and survey data were reviewed to guide iterative app refinements.

##### Portion size estimation usability and food selection accuracy (Final round only)

As part of the final round of testing, a subsample of participants completed a simulated food logging task designed to assess the usability of *Edna’s* portion size estimation workflow. Each participant was provided a standardized, one day menu comprising three meals and one snack. The foods were selected by the research team to reflect commonly consumed items in the U.S. diet and to represent a range of portion size and serving number combinations within the app. The same menu was used for all participants to ensure consistency of the task. Menus included AI-generated images created using ChatGPT (GPT-4, OpenAI) depicting complete meals presented in realistic place settings with standardized portions to simulate a plausible eating occasion (Supplementary Figure S1). Participants were provided only with the names and images of the foods; no actual meals were provided or consumed.

During a Zoom session, participants entered all menu items into *Edna* using their own devices and were instructed to select the portion size and number of servings that best reflected what they would typically consume in a similar situation, rather than matching the depicted portion. Participants were asked to think aloud while completing the task. Study staff observed the session but did not provide guidance on food or portion size selection. Because all participants had previously used *Edna* during earlier study phases, procedural questions were not anticipated. Following the simulation, participants completed a structured questionnaire to evaluate the usability of *Edna’s* portion size estimation interface. The survey included 5-point Likert-scale items assessing clarity and relevance of portion size images, ease of use, alignment with typical eating behaviors, and overall satisfaction. Additional open-ended items captured specific suggestions for improving the portion size flow, including terminology, labeling, and image presentation. The final item asked participants to rate how satisfied they would be if their suggested changes were implemented.

##### Participant compensation

Participants were compensated for their time and engagement, with compensation schemes varying slightly by study round. In Round 1, participants could earn up to $70, calculated as $5 per day of *Edna* use over the 14-day period, multiplied by their percentage of engagement. In subsequent rounds, compensation increased to a maximum of $80, including $1 for registering the app, $1 per day (up to 14 days) for logging foods and beverages consumed, $30 for completing the end-of-study feedback form, and $35 for completing the SUS. Participants who were randomized to complete the portion size testing task did not receive additional compensation. All payments were issued electronically after participation was verified.

#### Measures

##### Usability

The SUS was the primary quantitative usability outcome, and a predefined stopping rule was used to determine whether additional testing rounds were needed. The SUS, a validated 10-item questionnaire that yields a composite score from 0 to 100 on the general usability of a wide range of systems, including software, with higher scores indicating better perceived usability [26]. We planned for a minimum of two rounds of usability testing and app refinement with a goal of achieving an average SUS score of 80.8. Specifically, further rounds would be conducted only if SUS scores fell below a mean threshold of 80.8 in either sampling condition. This goal corresponds to a usability rating of A and above on the commonly used Sauro–Lewis SUS curved grading scale, indicating high user satisfaction and strong product usability [27]. Additionally, open-ended responses from an onboarding feedback survey, daily feedback surveys, end-of-study feedback form, and the portion size usability questionnaire were analyzed to identify usability barriers, navigation issues, and opportunities for improvement.

##### Engagement

Participant engagement was assessed using backend data from the *Edna* administrative dashboard. Engagement was defined as the percentage of the 14 study days on which a participant logged at least one food or beverage entry. This scheme-agnostic metric was used to characterize daily app use across both sampling conditions.

##### Interval completion (interval-contingent only)

For interval-contingent participants, interval completion was defined as the percentage of scheduled prompt intervals with any response (food entry or “Did not eat”). This metric reflects responsiveness to system-generated prompts and is reported as a feasibility indicator, not as a measure of complete dietary capture.

##### User satisfaction

Additional feedback on the satisfaction and usefulness of specific app features, including the onboarding tutorial, video tutorials, and the portion size selection process was obtained with 5-point Likert scales where higher scores reflected more favorable responses.

### Statistical analysis

#### Randomization and sample size justification

Participants were randomly assigned to event- or interval-contingent sampling using block randomization (block sizes 2, 4, or 6) generated with the Sealed Envelope simple randomizer (https://www.sealedenvelope.com/simple-randomiser/v1/lists) and implemented through REDCap at enrollment. Approximately 50 participants were enrolled per round, which provided 80% power to detect a moderate effect size (Cohen’s d ≈ 0.5) in SUS score comparisons between sampling conditions at α = 0.05.

#### Quantitative data analysis

Descriptive statistics were reported as means (SD) or frequencies (%). Between-round differences in participant characteristics were assessed using ANOVA for continuous variables and chi-square or exact tests for categorical variables, as appropriate. SUS scores and engagement were compared by sampling condition using independent samples t-tests and across rounds using one-way ANOVA. Linear regression models examined demographic and study-related predictors of SUS and engagement outcomes. Analyses were conducted in R (v4.2.2) using complete-case analysis.

#### Qualitative data analysis

Open-ended responses were analyzed using inductive thematic analysis. One study team member conducted initial line-by-line manual coding to identify recurring themes related to usability, navigation, and user experience. ChatGPT (GPT 5, OpenAI) coding was then applied as a secondary analytic pass to independently organize de-identified responses into thematic groupings and assess consistency of classification. Discrepancies between manual and AI-assisted categorizations were reviewed by the research team and resolved through discussion. Final theme assignments were determined by consensus. No identifiable participant information was entered into the AI system.

## Results

A total of 150 participants were enrolled in the study, distributed across multiple rounds of testing (Table 2). Those with fewer than 3 days of app use (*n* = 2) and/or who were missing SUS scores (*n* = 4) were excluded, resulting in an analytical sample of *N* = 146. Participants across the three rounds were generally representative of the U.S. population of adults aged 19–65 years with respect to age, Hispanic origin, and regional population estimates based on 2025 U.S. census estimates [28]. Females were slightly overrepresented at 57.5% compared to the national estimate of 50.5% and individuals with obesity were slightly underrepresented at 37.2% vs. 40.3% based on 2021–2023 NHANES data [29]. The sample was also representative of the U.S. race distribution with modest variations due to more participants identifying as a race other than the presented, single race options. No significant differences were observed across rounds.

**Table 2:** Participant characteristics by round (*N* = 146)

	All	Round 1	Round 2	Round 3	*P*-value^1^
*N*	146	46	45	55	
Age, years (mean (SD))	41.0 (12.7)	42.0 (14.2)	43.9 (12.6)	38.0 (11.0)	0.060
Sex (%)					0.698
Female	84 (57.5)	28 (60.9)	26 (57.8)	30 (54.5)	
Male	61 (41.8)	17 (37.0)	19 (42.2)	25 (45.5)	
Other	1 (0.7)	1 (2.2)	0 (0.0)	0 (0.0)	
Race (%)					0.632
White	105 (71.9)	36 (78.3)	31 (68.9)	38 (69.1)	
Black/African American	20 (13.7)	5 (10.9)	8 (17.8)	7 (12.7)	
American Indian/Alaskan Native	2 (1.4)	0 (0.0)	0 (0.0)	2 (3.6)	
Asian	1 (0.7)	0 (0.0)	1 (2.2)	0 (0.0)	
Native Hawaiian/Pacific Islander	1 (0.7)	0 (0.0)	1 (2.2)	0 (0.0)	
Other	17 (11.6)	5 (10.9)	4 (8.9)	8 (14.5)	
Ethnicity (%)					0.219
Hispanic or Latinx	32 (21.9)	6 (13.0)	10 (22.2)	16 (29.1)	
Non-Hispanic or Latinx	113 (77.4)	39 (84.8)	35 (77.8)	39 (70.9)	
Unknown	1 (0.7)	1 (2.2)	0 (0.0)	0 (0.0)	
BMI category (%)					0.196
18.5–24.9 kg/m^2^	44 (30.3)	14 (31.1)	14 (31.1)	16 (29.1)	
25–29.9 kg/m^2^	47 (32.4)	11 (24.4)	20 (44.4)	16 (29.1)	
≥ 30 kg/m^2^	54 (37.2)	20 (44.4)	11 (24.4)	23 (41.8)	
Education (%)					0.714
High school diploma, GED, or some college	21 (14.4)	6 (13.0)	6 (13.3)	9 (16.4)	
Associate’s or bachelor’s degree	64 (43.8)	17 (37.0)	22 (48.9)	25 (45.5)	
Graduate degree	61 (41.8)	23 (50.0)	17 (37.8)	21 (38.2)	
Partnered (%)	105 (71.9)	32 (69.6)	34 (75.6)	39 (70.9)	0.799
Geographic region (%)					0.873
Midwest	27 (18.5)	8 (17.4)	10 (22.2)	9 (16.4)	
Northeast	28 (19.2)	9 (19.6)	11 (24.4)	8 (14.5)	
Southeast	45 (30.8)	15 (32.6)	10 (22.2)	20 (36.4)	
Southwest	17 (11.6)	5 (10.9)	6 (13.3)	6 (10.9)	
West	29 (19.9)	9 (19.6)	8 (17.8)	12 (21.8)	

*SD* Standard deviation, *BMI* Body mass index, *GED* General Educational Development, P-values for between-round differences were calculated using ANOVA for continuous variables and Pearson’s chi-square test for categorical variables. Fisher’s exact test was applied to sex and race due to low cell counts. The underweight category was excluded from BMI analyses

Table 3 presents SUS scores and engagement by study round and sampling condition. Mean SUS scores increased across rounds, from 71.7 (SD = 23.0) in Round 1 to 78.8 (SD = 19.3) in Round 3. There were no significant differences in SUS scores between rounds (*p* = 0.187) or between the event- and interval-contingent groups within each round (all *p* ≥ 0.315).

**Table 3:** System Usability Scale (SUS) scores by round and sampling condition (*N* = 146)

	Round 1				Round 2				Round 3				
	All (*n* = 46)	Event (*n* = 24)	Interval (*n* = 22)	within round *p*-value	All (*n* = 45)	Event (*n* = 23)	Interval (*n* = 22)	within round *p*-value	All (*n* = 55)	Event (*n* = 25)	Interval (*n* = 30)	within round *p*-value	Between round *p*-value
Engagement (% days out of 14), mean (SD)	93.0 (17.8)	89.0 (22.6)	97.4 (9.2)	0.103	97.6 (7.5)	97.2 (9.3)	98.1 (5.0)	0.705	92.5 (15.6)	95.1 (9.2)	90.2 (19.2)	0.223	0.168
SUS Score, mean (SD)	71.7 (23.0)	72.2 (20.8)	71.2 (25.6)	0.893	77.6 (17.7)	77.0 (17.9)	78.3 (17.9)	0.803	78.8 (19.3)	81.6 (16.4)	76.4 (21.5)	0.315	0.187

Within-round p-values were calculated using independent samples t-tests. Between-round p-values were calculated using one-way ANOVA. SUS scores in Round 1 corresponded to a grade of C+ (range: 71.1–72.5). Scores in Rounds 2 and 3 correspond to a grade of B+ (range: 77.2–78.8) [23]. Round 3 event sampling met the predetermined threshold of ≥ 80.8

**Fig. 2:**
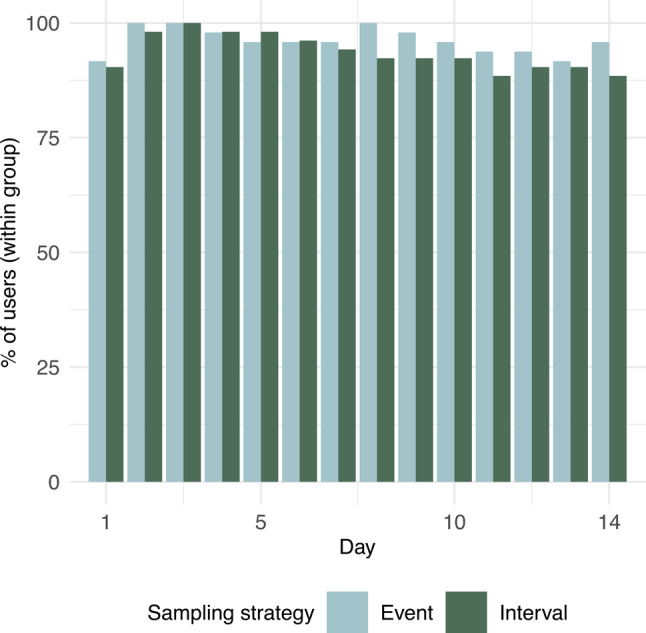
Daily engagement by sampling condition in rounds 2 and 3 (*N* = 100)

Mean engagement ranged from 92.5% (SD = 15.6%) in Round 3 to 97.6% (SD = 7.5%) in Round 2. Differences by round (*p* = 0.168) and sampling condition (all *p* ≥ 0.103) were not statistically significant. Daily engagement remained high across all 14 days for each sampling condition (Fig. 2, *p* = 0.306). Interval completion was assessed for participants in the interval-contingent group only. Prompt-level data were unavailable in Round 1. In Round 2, mean interval completion was 87.4% (SD = 15.0%), and in Round 3, it was 76.0% (SD = 24.5%).

In the regression model, sex and education were the only significant predictors of SUS scores. Males rated usability an average of 7.3 points higher than females (95% CI: 0.4 to 14.2, *p* = 0.038). Participants with a graduate degree rated usability 13.3 points lower than those with less education (95% CI: −23.4 to − 3.2, *p* = 0.010). SUS scores did not differ significantly by age, race, ethnicity, BMI category, or U.S. region. There were no significant differences in engagement scores by any demographic or study-related variable.

### Qualitative findings and *Edna* modifications

Table 4 presents the most frequently cited usability issues reported by participants, along with illustrative feedback and the corresponding refinements made across development rounds. Common challenges included confusion around food categorization, the absence of portion sizes, and difficulties locating specific foods. Many users also expressed a desire for more guidance, especially early in the logging process, and reported issues related to app stability, editing entries, and discovering key features.

**Table 4:** Top cited usability issues and refinement decisions

Usability Issue	Description	Example User Feedback	Refinement Made
Limited food options	Users requested more food variety, especially for cultural dishes, restaurant meals, and add-ons.	“Include more diverse food options. I wanted to add a taco bowl or Korean BBQ.”	Reviewed missing foods and updated list; added aliases for difficult-to-find items (after rounds 1 and 2).
Confusing food categorization	Users struggled to locate foods in the list and didn’t always understand how to browse categories.	“Some foods were hard to find. For example, I expected to find burgers under fast food, not sandwiches.”	Introduced “Find My Food” feature with crumb trail (after round 1); reworded some category labels (after rounds 1 and 2).
Desire for more guidance	Participants wanted more support during food entry, especially at the beginning.	“A guide to help during the first couple of days would be helpful.”	Created multiple new tutorial videos (e.g. complex meals, how to find foods) (after round 1).
Bugs and crashes	Users reported app freezing, crashing, or being slow to load.	“App shuts down a lot.” “Sometimes freezes, have to exit and re-open.”	Addressed crashes and bugs between rounds; improved app stability and speed (after rounds 1 and 2).
Want to edit or add to events	Users found it frustrating to start a new flow to add foods they forgot earlier.	“I forgot to add a beverage to a meal but couldn’t just add it.”	Enabled adding new foods to existing events without repeating the full entry process (after round 2).
Calendar and feedback visibility	Users wanted to know which days had been completed and see an overview of entries.	“Would be nice to see what days I’ve logged.”	Updated calendar to show days with entries; added daily summary screen (after round 1).
Time entry limitations	Some users wanted to log the exact time they ate, rather than choosing from intervals.	“Allow me to put in the actual time I ate.”	Switched from 15-minute intervals to clock-time entry (after round 2).
Reminders and prompts	Users missed some features because they didn’t know where to find them or forgot they existed.	“Didn’t know I could favorite foods.”	Added 24-hour prompts after first use to highlight FAQs, videos, Favorites, and Find My Food (after round 2).

Between rounds 1 and 2, *Edna* was refined to improve stability, clarity, and ease of use. Technical issues were addressed, and onboarding was updated to clearly explain the app’s purpose (i.e., focus on specific foods high in saturated fat or added sugar to support research). In parallel, tutorial videos and FAQs were expanded, and interface updates included improvements to previewing food items, a daily summary screen, and a visual calendar showing which days had entries. To reduce the number of difficult to find foods, *Edna* was improved in 3 ways: (1) food category labels were revised to highlight the location of foods, (2) the “Find My Food” feature was introduced as an in-app navigation assistance feature, and (3) food aliases for cultural and regional foods (e.g., “pad Thai” = “Asian noodle dish”) that were embedded into the “Find My Food” feature.

Between rounds 2 and 3, changes focused on addressing the most persistent issues. Feature visibility was improved through contextual prompts after the first 24 h of app use, and the food entry flow was revised to allow users to add items to existing meals without restarting the process. Additional, minor refinements included new food aliases and allowing users to enter exact clock times for meals.

Improvements made between rounds 1 and 2 resulted in the greatest observed improvements in usability with the highest SUS score in the final round of testing (Table 3). Similarly, the greatest reduction in the approximate number of difficult to find foods was observed between rounds 1 and 2 (Round 1: 100; Round 2: 62; Round 3: 65). The limited improvements in usability achieved between rounds 2 and 3, confirmed that additional rounds of refinements would no longer appreciably improve usability scores.

### Portion size estimation task and *Edna* modifications

Of the 55 participants in Round 3, *n* = 34/55 (61.8%) volunteered to provide feedback on the portion size selection demo in the app. Overall impressions were positive. Most participants, *n* = 32/34 (94.1%), rated their overall experience using the app, including portion size selection, as *excellent* or *nearly excellent*. Satisfaction with the portion size selection process was also high, with *n* = 22/34 (64.7%) reporting they were *very satisfied* or *satisfied*.

Portion size labels were generally clear: *n* = 30/34 (88.2%) reported no foods with unclear or confusing labels. Selecting portion sizes required *very little* or *little* effort for *n* = 26/34 (76.5%), and all participants, *n* = 34/34 (100%), found the labels shown with portion size images (e.g., “small,” “medium,” “1 cup”) to be *understandable* or *very understandable*. Most participants (*n* = 22/34, 64.7%) thought that the portion size options were well matched to the amounts of food they typically eat.

Qualitative feedback supported the positive quantitative findings. Most described the portion size selection process as “very easy to navigate” and “straightforward,” with several highlighting the usefulness of visual cues. One participant noted, “Seeing a visual was very helpful. Also having different options like ‘1 cup’ or ‘1 container’ made it feel easy.” Many reported no difficulties, while a few identified specific foods that were challenging to interpret. For example, one participant remarked that “cupcake portion pics included icing, but icing was available as an add-on”. Suggestions for improvement were minimal, and most participants provided no additional dislikes. Participant feedback informed the final design of *Edna’s* image-based portion size selection feature. The success of these design refinements was evident in that participants who had previously experienced common issues (e.g., entering the portion size for eggs) no longer encountered problems with the refined version.

## Discussion

This study evaluated the usability of *Edna*, an mEMDA platform developed to modernize population-level dietary surveillance. Across iterative rounds of national testing, *Edna* achieved above-average usability and strong engagement, demonstrating that mobile EMA can feasibly collect detailed dietary data across diverse adults. Participants rated the interface as intuitive and efficient, and engagement remained high throughout daily use, supporting proof-of-concept for scalable, image-supported dietary surveillance.

Results reinforced the feasibility of EMA-based dietary assessment. SUS scores rose from 71.7 to 78.8 in three rounds of iterative design and usability testing. These scores are above the widely accepted “average” benchmark of 68 for digital tools and approached the range typical of well-designed health applications [30, 31]. Similarly, participants rated the image-assisted portion module highly for clarity and ease. These findings are consistent with prior evidence that image guidance improves portion estimation and reduces burden [32]. The most substantial improvement to the SUS scores occurred between Rounds 1 and 2, coinciding with refinements to the leveled food-entry flow: simplifying food categorization, introducing a “Find My Food” helper with aliases and synonyms, enabling adding foods to existing meals, improving clock-time entry and calendar navigation, and surfacing Favorites for one-tap re-entry. These adjustments directly addressed early user feedback about navigation and edit burden, producing measurable gains in perceived usability. SUS scores plateaued after Round 2, suggesting that the interface had reached a stable and satisfactory level of usability where further gains would likely require micro-interaction or aesthetic refinements rather than structural redesign, despite falling short of the pre-defined A grade usability goal of 80.8 on the SUS.

Engagement was high (≈ 90%) across rounds. Interval completion ranged from 76% to 87% across rounds. These values are comparable to or higher than published EMA benchmarks, as meta-analytic findings place overall EMA compliance near 75% in substance-use studies [33] and around 79% in general mobile-EMA designs [34]. However, engagement and interval completion should be interpreted in light of the tiered incentive structure used in this formative study, which rewarded prompt responsiveness and may have contributed to elevated participation rates. Usability, engagement, and interval completion were generally similar across event- and interval-contingent protocols. While these findings require confirmation under stable platform conditions and in free-living settings, they suggest that sampling-scheme selection in future studies may be guided by analytic priorities, such as capturing contextual richness versus estimating time-stamped prevalence, rather than by concerns about differential burden alone.

Strengths of the usability study include a priori usability benchmarks, randomized comparison of sampling protocols, national remote recruitment, and a multimethod approach linking concrete design refinements to measurable usability gains. *Edna’s* food list was derived from nationally representative NHANES data and usability testing included a diverse U.S. adult sample, supporting generalizability to adults aged 19 to 65 years. Findings may not extend beyond English-speaking U.S. smartphone users. Additional limitations warrant consideration. Participants were compensated, which may influence engagement, although compensation is standard in EMA research and applied consistently across conditions [35]. This manuscript reports formative usability findings rather than definitive evaluation of dietary reporting completeness or sampling-scheme performance. Engagement and interval completion reflect app use and prompt responsiveness but do not verify comprehensive capture of eating occasions, and prompt-level completion cannot be estimated for the self-initiated event-contingent condition. Because app features evolved across rounds, cross-round comparisons should be interpreted cautiously.

## Conclusions

Collectively, these findings support the feasibility and acceptability of mobile ecological momentary diet assessment for population-level dietary surveillance when grounded in iterative, user-centered design. By combining real-time self-report, visual portion guidance, and automated nutrient coding within an accessible interface, *Edna* provides a practical bridge between traditional 24-hour dietary recalls and emerging digital surveillance methods. As public health agencies seek scalable approaches that capture diet more dynamically and inclusively, *Edna* offers a foundation for integrating mEMDA into national nutrition monitoring systems to strengthen evidence-based efforts to improve diet quality and reduce nutrition-related inequities.

## Future directions

Overall usability was strong; however, a few participants indicated that the leveled food-entry flow could be further streamlined. To modernize *Edna’s* interface and enhance efficiency, future development efforts will integrate AI-assisted search and adaptive prompting to make food identification faster and more intuitive. The next phase will validate *Edna* against known foods to quantify accuracy in food identification and portion selection and conduct a free-living comparison of event- versus interval-contingent sampling to evaluate reporting accuracy, prompt responsiveness, and recall fidelity under naturalistic conditions. These efforts will extend *Edna’s* capability as a scalable, user-centered platform for modern dietary surveillance.

## Supplementary Information

Supplementary Material 1.

## Data Availability

The datasets generated and analyzed during the current study are available in the Zenodo repository, 10.5281/zenodo.17538030.

## Abbreviations

24HR: 24-hour dietary recall
BMI: Body mass index
EMA: Ecological momentary assessment
FFQ: Food frequency questionnaire
FNDDS: Food and Nutrient Database for Dietary Studies
mEMDA: Mobile ecological momentary diet assessment
NHANES: National Health and Nutrition Examination Survey
NIH: National Institutes of Health
SUS: System Usability Scale
USDA: United States Department of Agriculture
