# Supplementary figures and images for: Development and usability of a mobile ecological momentary assessment platform for dietary surveillance in the U.S
Source: Int J Behav Nutr Phys Act. 2026 Apr 25;23:61. doi: 10.1186/s12966-026-01916-x (PMC13251139; doi:10.1186/s12966-026-01916-x)

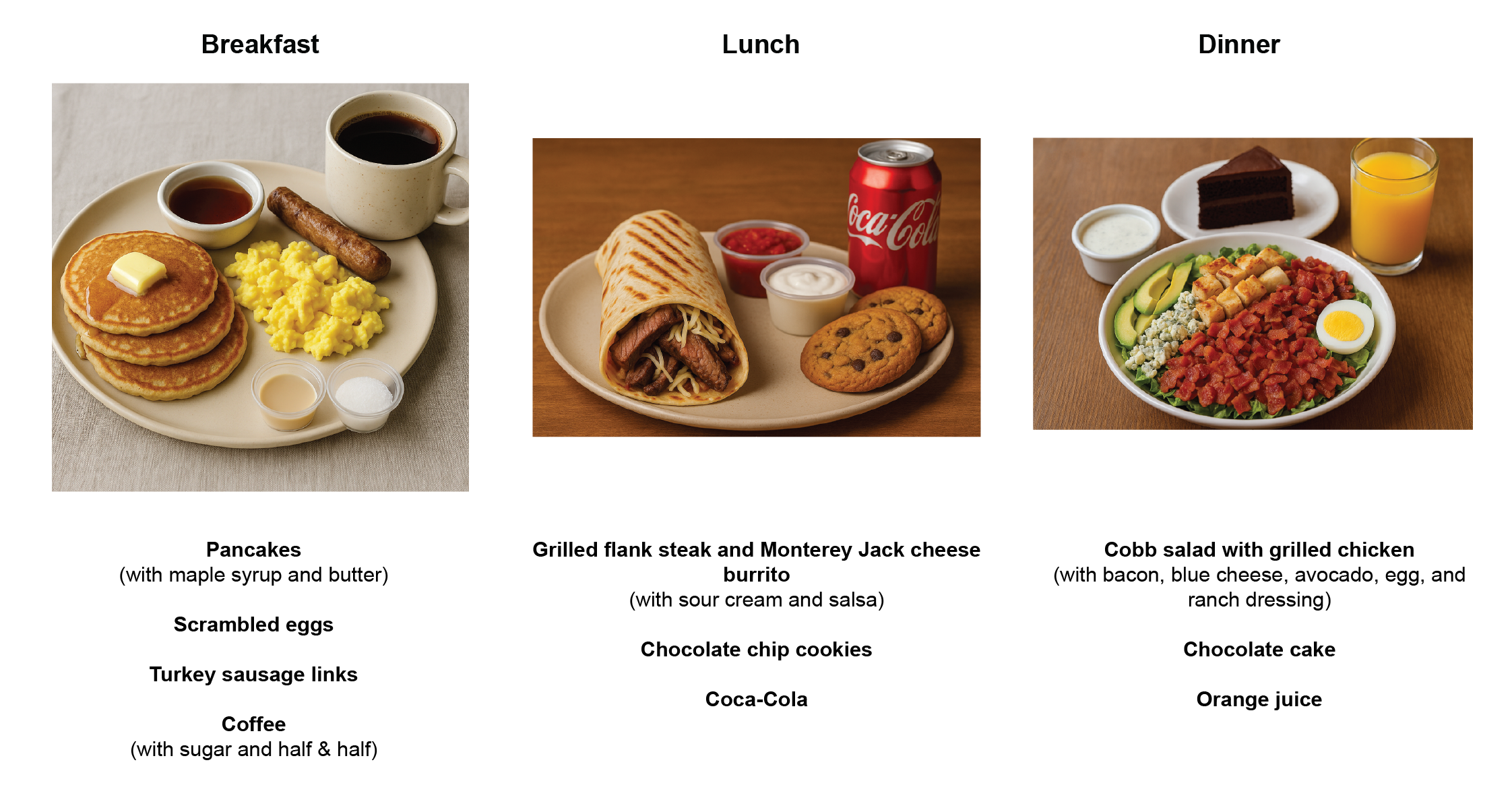

Supplement: Supplementary file 1 — Supplementary Material 1. [file 12966_2026_1916_MOESM1_ESM.png]
